# Association of Fine Particulate Matter and Risk of Stroke in Patients With Atrial Fibrillation

**DOI:** 10.1001/jamanetworkopen.2020.11760

**Published:** 2020-09-15

**Authors:** Zachary J. Rhinehart, Ellen Kinnee, Utibe R. Essien, Melissa Saul, Emily Guhl, Jane E. Clougherty, Jared W. Magnani

**Affiliations:** 1 Department of Medicine, University of Pittsburgh, Pittsburgh, Pennsylvania; 2 University Center for Social and Urban Research, University of Pittsburgh, Pittsburgh, Pennsylvania; 3 Center for Health Equity Research and Promotion, Veterans Affairs Pittsburgh Healthcare System, Pittsburgh, Pennsylvania; 4 Department of Environmental and Occupational Health, Dornsife School of Public Health, Drexel University, Philadelphia, Pennsylvania

## Abstract

**Importance:**

Air pollution is associated with cardiovascular outcomes. Specifically, fine particulate matter measuring 2.5 μm or less (PM_2.5_) is associated with thrombosis, stroke, and myocardial infarction. Few studies have examined particulate matter and stroke risk in individuals with atrial fibrillation (AF).

**Objective:**

To assess the association of residential-level pollution exposure in 1 year and ischemic stroke in individuals with AF.

**Design, Setting, and Participants:**

This cohort study included 31 414 individuals with AF from a large regional health care system in an area with historically high industrial pollution. All participants had valid residential addresses for geocoding and ascertainment of neighborhood-level income and educational level. Participants were studied from January 1, 2007, through September 30, 2015, with prospective follow-up through December 1, 2017. Data analysis was performed from March 14, 2018, to October 9, 2019.

**Exposures:**

Exposure to PM_2.5_ ascertained using geocoding of addresses and fine-scale air pollution exposure surfaces derived from a spatial saturation monitoring campaign and land-use regression modeling. Exposure to PM_2.5_ was estimated annually across the study period at the residence level.

**Main Outcomes and Measures:**

Multivariable-adjusted stroke risk by quartile of residence-level and annual PM_2.5_ exposure.

**Results:**

The cohort included 31 414 individuals (15 813 [50.3%] female; mean [SD] age, 74.4 [13.5] years), with a median follow-up of 3.5 years (interquartile range, 1.6-5.8 years). The mean (SD) annual PM_2.5_ exposure was 10.6 (0.7) μg/m^3^. A 1-SD increase in PM_2.5_ was associated with a greater risk of stroke after both adjustment for demographic and clinical variables (hazard ratio [HR], 1.08; 95% CI, 1.03-1.14) and multivariable adjustment that included neighborhood-level income and educational level (HR, 1.07; 95% CI, 1.00-1.14). The highest quartile of PM_2.5_ exposure had an increased risk of stroke relative to the first quartile (HR, 1.36; 95% CI, 1.18-1.58). After adjustment for clinical covariates, income, and educational level, risk of stroke remained greater for the highest quartile of exposure relative to the first quartile (HR, 1.21; 95% CI, 1.01-1.45).

**Conclusions and Relevance:**

This large cohort study of individuals with AF identified associations between PM_2.5_ and risk of ischemic stroke. The results suggest an association between fine particulate air pollution and cardiovascular disease and outcomes.

## Key Points

**Question:**

What is the association between fine particulate matter measuring 2.5 μm or less (PM_2.5_) air pollution and ischemic stroke in individuals with prevalent atrial fibrillation (AF)?

**Findings:**

This cohort study including 31 414 individuals with AF found an association between PM_2.5_ and prospective ischemic stroke risk in longitudinal, residential-level assessments in a large health care system situated in a region with high industrial activity. In multivariable-adjusted analyses that included relevant covariates and neighborhood-level income and educational level, individuals in the highest quartile of PM_2.5_ exposure had a 1.2-fold higher risk of stroke compared with the lowest quartile.

**Meaning:**

The association between residential-level pollution and stroke risk in the presence of AF appears to be an additional public health toll of pollution and suggests that stroke risk assessment in individuals with AF take into account the contributions of environmental exposures.

## Teaser

This cohort study assesses the association between annual, residential-level exposure to fine particulate matter and ischemic stroke in individuals with atrial fibrillation.

## Introduction

Atrial fibrillation (AF) is a common heart rhythm disorder, and thromboembolic stroke is a chief associated outcome.^1,2^ Risk factors for stroke in individuals with AF are well established.^3^ In contrast, how environmental exposures augment ischemic stroke risks in AF remains unexplored. Such an investigation has the potential to elucidate mechanisms of stroke pathogenesis in AF and facilitate individualized approaches to care that incorporate the reduction of pollution exposure to modify stroke risk.

**Figure 1.**
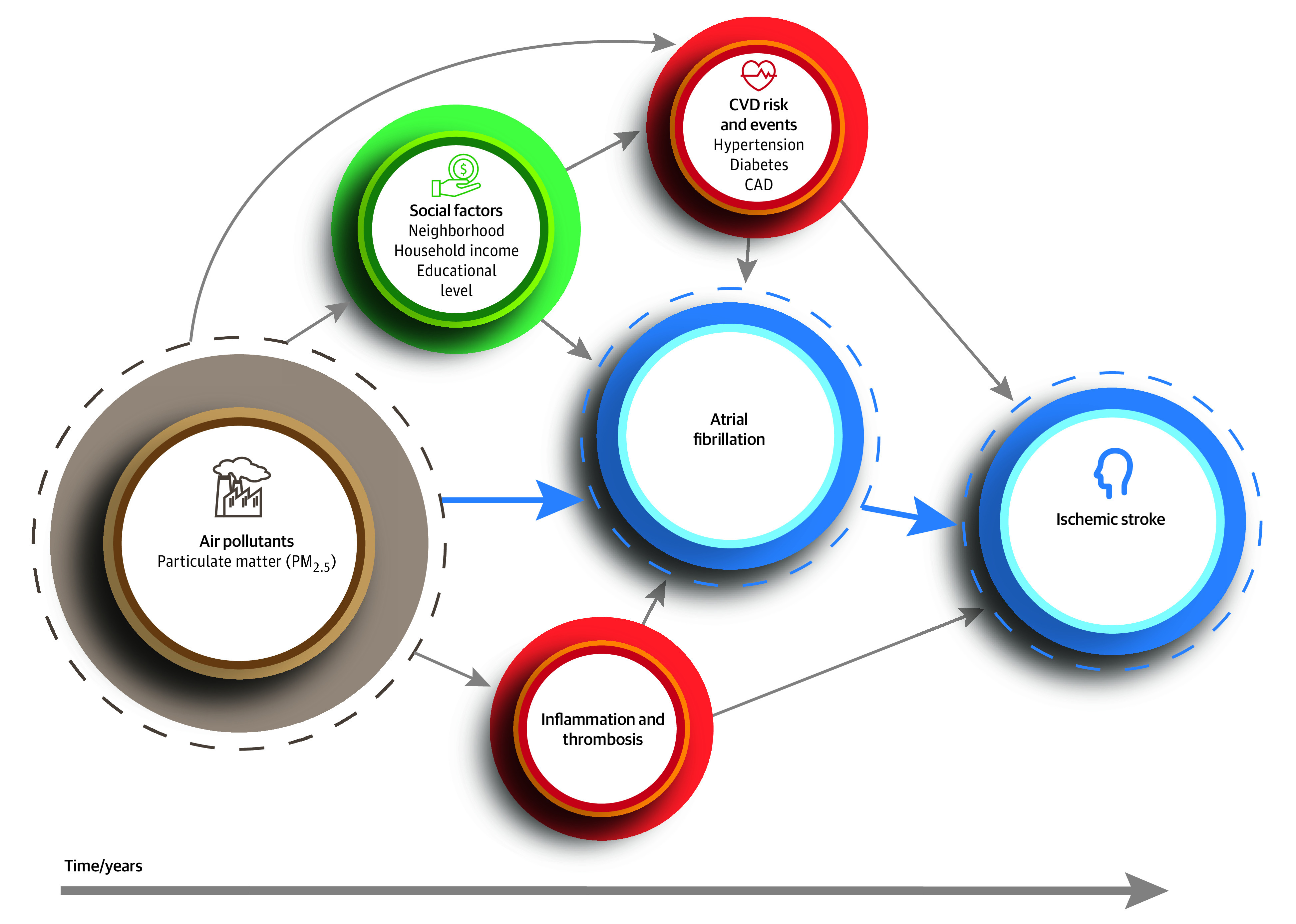
A Pathway for the Association Between Particulate Matter and Atrial Fibrillation and Risk of Ischemic Stroke CAD indicates coronary artery disease; CVD, cardiovascular disease; and PM_2.5_, fine particulate matter measuring 2.5 μm or less. The dashed lines indicate the main components examined and the solid-blue arrows how they are related.

Ambient air pollutants, especially fine particulate matter measuring 2.5 μm or less (PM_2.5_), are associated with cardiovascular risk.^4^ Multiple studies^5,6,7,8,9,10^ have established the associations between short-term (diurnal) and long-term (multiyear) PM_2.5_ exposures with cardiovascular events. PM_2.5_ has been identified as having a causal relation to cardiovascular disease and being the foremost environmental risk factor for cardiovascular events,^4,11^ including stroke and stroke-related mortality.^12,13,14,15,16^ A population-based study^17^ found that long-term pollution augments stroke risk in individuals with stroke risk factors. In Figure 1, we present a pathway by which particulate matter may be associated with AF and stroke. Because prior studies^18,19^ have focused on short-term pollution changes and AF hospitalization events, we examined the long-term, residence-specific association of pollution, specifically PM_2.5_, with risk of stroke in patients with AF.

Examining the association between environmental pollutant exposures and long-term stroke events in individuals with AF requires a combination of detailed assessments of exposure (ie, precise spatial and temporal measurement of pollutants across a large area) and determination of individual-level clinical outcomes (as collected by a regional health care system). A prior study^20^ investigated stroke without considering AF. A major question remains as to how long-term pollution exposure is associated with stroke risk in AF.

We used data from a spatial saturation air pollution monitoring campaign in Allegheny County, located in southwestern Pennsylvania, a region with a history of heavy industrial activity. Pittsburgh, Pennsylvania, the largest city in Allegheny County, was ranked by the American Lung Association’s 2019 State of the Air report as 1 of the 10 most polluted cities in the US.^21^ We combined electronic health data from the region’s largest health care system with annual, residential-level assessments of particulate matter exposure. We hypothesized that PM_2.5_ exposure has a dose-dependent association with stroke risk in patients with AF, such that individuals with greater long-term PM_2.5_ exposure would have a correspondingly increased stroke risk.

## Methods

### Study Cohort and Ascertainment of AF

We performed a cohort study of individuals with AF identified from a regional health care system with a uniform electronic health record (EHR) from January 1, 2007, to September 30, 2015. We geocoded individual addresses to assign annual estimates of residential-level particulate matter exposure. The University of Pittsburgh Human Research Protection Office approved this study as minimal risk research and waived informed consent. We followed the requirements of the Strengthening the Reporting of Observational Studies in Epidemiology (STROBE) reporting guideline.^22^

The University of Pittsburgh Medical Center (UPMC) provides 41% of health services to the Western Pennsylvania region, with a patient volume in 2018 that exceeded 388 000 admissions and 5.5 million outpatient visits.^23^ The UPMC EHR systems are stored in the Medical Archival System (MARS), a repository for the health system’s electronic clinical, administrative, and financial databases that was developed at the University of Pittsburgh.^24^

We searched MARS to identify individuals seen at the UPMC between January 1, 2007, and September 30, 2015, who were residing in Allegheny County, Pennsylvania, and had a diagnosis of AF determined on electrocardiogram, ablation, or cardioversion procedure for AF or at least 2 separate encounters with *International Classification of Disease, Ninth Edition* (*ICD-9*) codes for AF (eg, code 427.31).^25^ A total cohort of 41 002 was identified. We defined the start of follow-up as the date at which these criteria were met.

We selected 200 cases randomly across study years to verify AF. Two physicians (Z.J.R. and U.R.E.) independently reviewed the EHR for each case with a third assigned to adjudicate (E.G.). We confirmed 198 of the 200 cases as AF. Two of the 198 confirmed cases required adjudication by a third reviewer (E.G.) for confirmation of AF.

We excluded 7 individuals younger than 18 years, 2253 with a history of ischemic stroke before the diagnosis of AF, and 3660 who underwent cardiothoracic surgery within 30 days of AF diagnosis. We further excluded 547 individuals without UPMC follow-up after determination of AF. eFigure 1 in the Supplement summarizes cohort selection by stepwise exclusion.

### Address Geocoding

We extracted the home address for each participant from the EHR and geocoded to x,y coordinates in ArcGIS (Environmental Systems Research Institute) using a composite address locator to maximize the positional accuracy of the address location. For our geocoding protocol, we (1) excluded incomplete addresses (eg, post office boxes and those of participants who were undomiciled); (2) ran addresses through a US Postal Service reference data set using ZP4 address standardization software (Semaphore Corporation); (3) excluded addresses outside Allegheny County; and then (4) sequentially geocoded addresses with an address point locator, a parcel layer locator, and a street network locator.^26,27^ We excluded 2234 individuals with addresses that could not be geocoded. The remaining 31 414 addresses were geocoded with a 97.2% match rate. We removed addresses from the analytic data set after geocoding for protection of the study participants.

### Particulate Matter Exposure

The primary independent variable was annual mean exposure to PM_2.5_ estimated at individual residential location. Our approach toward PM_2.5_ quantification is well detailed elsewhere.^28^ In brief, we conducted a spatial-saturation monitoring campaign at 37 distinct sites during summer (June to July 2012) and winter (January to March 2013) across a region of approximately 388 km^2^. At each site, integrated PM_2.5_ samples were collected using Harvard Impactors (Air Diagnostics and Engineering Inc), mounted at 10 to 12 ft, operated at a flow rate of 4.01 L/min, for the first 15 minutes of each hour per season during a 7-day sampling period. Sampling sites were selected with geographic information systems (GISs) to capture spatial variation and differences in traffic density, proximity to industry, and elevation. A land use regression (LUR) modeling approach was used to model PM_2.5_ concentrations as a function of GIS-based indicators of pollution sources and land use characteristics (eg, traffic density; transportation networks; roadway; industrial emissions; population; and truck, bus, and diesel indicators), accounting for temporal variation using concentrations at a reference monitoring site.^28,29^ Using the LUR models, we created a continuous spatial surface of estimated PM_2.5_ and used that surface to estimate 1-year mean exposures within the 300-m buffer that surrounded each participant’s home.^29,30^ Earlier work^31^ has demonstrated stable spatial variance in PM_2.5_ concentrations, with the same areas remaining relatively high or low for years. Therefore, estimating exposure based on residual location effectively identifies individuals with consistently higher or lower exposures over time. Consequently, we assigned a single annual mean air pollution measure as the exposure to each residence during the study period.

### Ischemic Stroke

Our primary outcome was hospitalization for ischemic stroke, defined as a hospitalization event with primary diagnosis of ischemic stroke by administrative coding.^32^ We defined the date of stroke as the initial date of hospitalization. The time to event was determined as the start of observation (ie, earliest identification of AF in the EHR) to the first stroke event during the observation period. Individuals were followed up prospectively for stroke events to December 1, 2017.

### Covariates

We searched MARS for patient-level demographic information (sex and race) and *ICD-9* codes for comorbid medical conditions and outcomes (eTable 1 in the Supplement). Comorbid medical conditions were selected by their established associations with increased ischemic stroke risk in individuals with AF: heart failure, hypertension, diabetes, coronary artery and peripheral vascular disease, and transient ischemic attack.^3^ We considered a comorbidity present if there was an *ICD-9* diagnosis before or at the start of participant observation. We used a GIS to derive neighborhood-level socioeconomic characteristics from the US Census Bureau’s American Community Survey from 2011 to 2015.^33^ We included neighborhood median household income and percentage of census tract residents with a high school diploma and bachelor’s degree as covariates.

### Statistical Analysis

We report continuous variables with normal distributions as mean (SD) and those deviating from normal distributions as median (interquartile range [IQR]). We compared continuous variables using 2-tailed, independent-sample *t* tests, Wilcoxon-Mann-Whitney tests, and Spearman correlations. We tested categorical variables using the Fisher exact or χ^2^ tests. Follow-up for each participant was censored at the date of the last EHR record available, including mortality, or at 10 years of observation. We determined incidence rates for ischemic stroke by quartile of PM_2.5_. Kaplan-Meier estimates were created to describe ischemic stroke events by quartile of PM_2.5_ exposure. We used Cox proportional hazards regression models to estimate the hazard ratio (HR) and 95% CI for the time to ischemic stroke associated with a 1-SD increase in PM_2.5_ and then for each quartile of PM_2.5_ exposure with the lowest quartile as the referent. We controlled for the nonlinear association between age and stroke risk using quadratic terms (age squared and age cubed).^34,35^ We adjusted models for sex, race (Black vs not Black), and the established stroke risk factors. We excluded transient ischemic attack from multivariable adjustment, given the limited specificity of the diagnosis.^36^ We tested the proportional hazards assumption for each covariate and adjusted the model to include time-dependent covariates for each variable that violated this assumption (age, sex, history of heart failure, and history of hypertension). We used a 2-sided α = .05 to determine statistical significance. Data analysis was performed from March 14, 2018, to October 9, 2019. All analyses were performed using Stata SE software, version 13.1 (StataCorp LLC).

## Results

**Figure 2.**
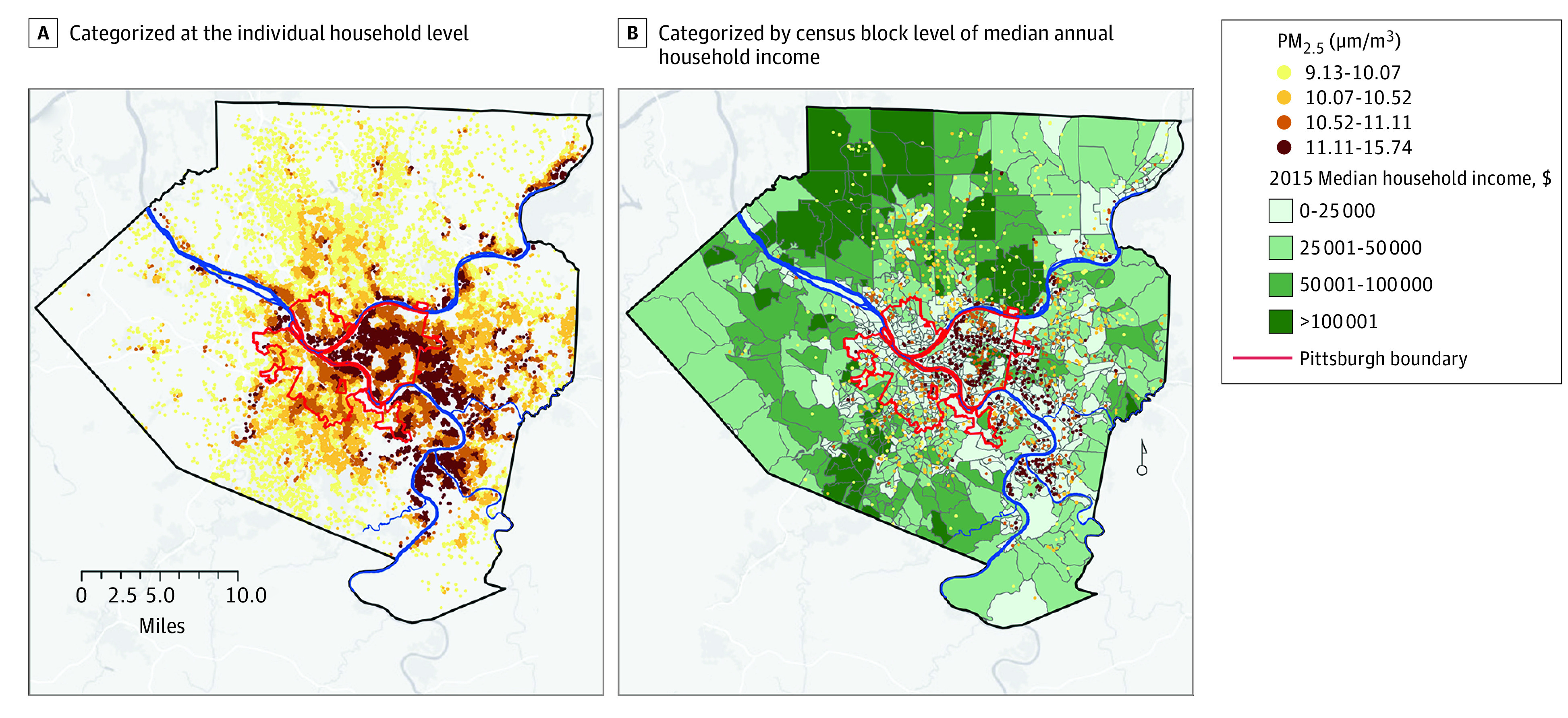
Map of Allegheny County, Pennsylvania The map shows fine particulate matter measuring 2.5 μm or less (PM_2.5_) concentration, categorized by quartile at the residence level for (A) each cohort participant (n = 31 414) and (B) for each cohort participant with a stroke event (n = 1546) and by census block level of median household income. PM_2.5_ values appear to overlap because of rounding.

After exclusions, 31 414 individuals (15 813 [50.3%] female; mean [SD] age, 74.4. [13.5] years) were included in the study cohort. Table 1 summarizes characteristics by quartile of PM_2.5_ exposure. Medical comorbidities and stroke risk factors were highly prevalent: heart failure (17 748 [56.5%]), hypertension (25 622 [81.6%]), diabetes (10 011 [31.9%]), and coronary artery disease (13 657 [43.5%]). The mean (SD) annual PM_2.5_ exposure was 10.6 (0.7) μg/m^3^. Figure 2A presents the residential-level annual PM_2.5_ estimate, demonstrating spatial clustering of pollution exposure across the study region (Allegheny County, Pennsylvania).

**Table 1.** Baseline Characteristics and Comorbidities According to PM_2.5_ Quartile^a^

Characteristic	Quartile 1 (PM_2.5_ range, 9.13-10.07 μg/m^3^) (n = 7856)	Quartile 2 (PM_2.5_ range, 10.07-10.52 μg/m^3^) (n = 7915)	Quartile 3 (PM_2.5_ range, 10.52-11.11 μg/m^3^) (n = 7847)	Quartile 4 (PM_2.5_ range, 11.11-15.74 μg/m^3^) (n = 7796)
Age, median (IQR), y	76.6 (66.2-84.4)	77.7 (67.4-85.0)	76.9 (65.3-84.4)	76.6 (64.6-85.0)
Female sex	3616 (46.0)	3968 (50.1)	4038 (51.5)	4191 (53.8)
Black race	75 (1.0)	330 (4.2)	936 (11.9)	1572 (20.2)
Heart failure	3935 (50.1)	4350 (55.0)	4613 (58.8)	4850 (62.2)
Hypertension	6144 (78.2)	6363 (80.4)	6497 (82.8)	6618 (84.9)
Diabetes	2089 (26.6)	2432 (30.7)	2645 (33.7)	2845 (36.5)
CAD	3002 (38.2)	3310 (41.8)	3545 (45.2)	3800 (48.7)
PAD	831 (10.6)	982 (12.4)	1081 (13.8)	1238 (15.9)
TIA	219 (2.8)	255 (3.2)	248 (3.2)	235 (3.0)
Social factors, median (IQR)^b^				
Annual household income, $	78 021 (58 913-96 510)	56 069 (46 132-73 588)	44 319 (35 873-56 148)	33 166 (25 467-43 386)
Some college attendance, %	14.8 (10.4-18.1)	16.5 (12.7-21.2)	18.4 (12.6-23.9)	18.4 (11.8-24.5)
High school diploma, %	23.7 (16.6-30.8)	28.4 (22.9-37.5)	30.5 (21.2-39.1)	31.3 (19.5-39.1)

Abbreviations: CAD, coronary artery disease; IQR, interquartile range; PAD, peripheral arterial disease; PM_2.5_, particulate matter measuring 2.5 μm or less; TIA, transient ischemic attack.

^a^ Data are presented as number (percentage) of participants unless otherwise indicated.

^b^ Social factors derived from estimates obtained by the US Census Bureau.^33^

The median follow-up time in the cohort was 3.5 years (IQR, 1.6-5.8 years), with a total observation time of 122 745 person-years. During this time, 1546 patients had an ischemic stroke, with an overall event rate of 12.60 per 1000 person-years (95% CI, 11.98-13.24). A 1-SD increase in PM_2.5_ was associated with an increased risk of stroke (HR, 1.08; 95% CI, 1.03-1.14) with adjustment for demographic and clinical variables. With full multivariable adjustment, including household income and educational level, a 1-SD increase in PM_2.5_ was associated with an increased risk of stroke (HR, 1.07; 95% CI, 1.00-1.14).

eTable 2 in the Supplement gives the event rates for ischemic stroke per 1000 person-years according to PM_2.5_ quartile stratified by age and sex. The data show a positive correlation between the incidence of ischemic stroke and quartile of residential-level estimates of PM_2.5_. In age- (<75 and ≥75 years) and sex-stratified analyses, the incidence of ischemic stroke remained elevated with increased PM_2.5_ exposure. The Kaplan-Meier curves for PM_2.5_ in age-stratified analyses are shown in eFigure 2A and B in the Supplement and in sex-specific strata in eFigure 3A and B in the Supplement. These supplementary figures illustrate the consistent association of residential level PM_2.5_ across age- and sex-specific strata.

**Figure 3.**
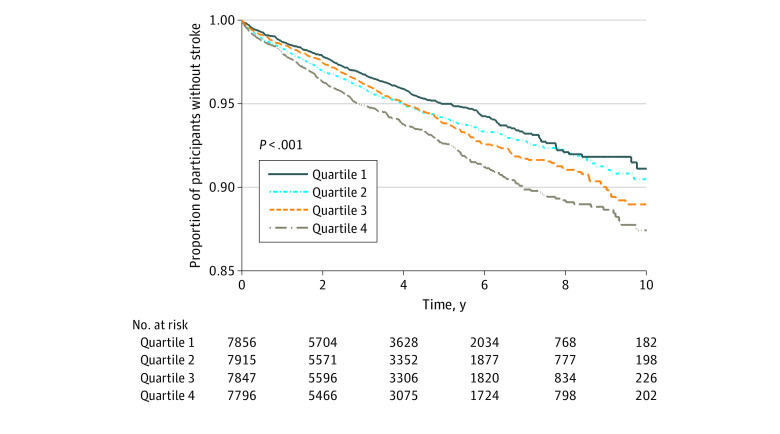
Kaplan-Meier Curve Showing Ischemic Stroke Events During Observation Years by Quartile of Particulate Matter Measuring 2.5 μm or Less (PM_2.5_) The figure demonstrates the long-term associations of residential-level estimates of PM_2.5_ with stroke events.

In analysis adjusted for age, sex, and race, the highest quartile of PM_2.5_ exposure was associated with increased risk of stroke when compared with the lowest (HR, 1.36; 95% CI, 1.18-1.58) (Table 2). After multivariable adjustment that included the clinical covariates and neighborhood-level income and educational level, the association between PM_2.5_ exposure and stroke risk for the highest quartile was attenuated to an HR of 1.21 (95% CI, 1.01-1.45) compared with the lowest quartile referent. Figure 2B shows stroke events by PM_2.5_ quartile with census block–level estimates of median annual household income, demonstrating clustering of stroke cases conjoint with this social factor. Figure 3 presents the Kaplan-Meier curves for stroke events over time by PM_2.5_ quartile, demonstrating the long-term association of residential estimates of PM_2.5_ with increased stroke events.

**Table 2.** Association of PM_2.5_ by Quartile and Risk of Stroke^a^

PM_2.5_ quartile	HR (95% CI)	*P* value
Model 1		
1	1 [Reference]	NA
2	1.12 (0.97-1.30)	.13
3	1.15 (0.99-1.34)	.06
4	1.36 (1.18-1.58)	<.001
Model 2		
1	1 [Reference]	NA
2	1.07 (0.92-1.24)	.40
3	1.08 (0.93-1.25)	.33
4	1.25 (1.08-1.45)	.003
Model 3		
1	1 [Reference]	NA
2	1.06 (0.91-1.24)	.46
3	1.05 (0.89-1.24)	.54
4	1.21 (1.01-1.45)	.04

Abbreviations: HR, hazard ratio; NA, not applicable; PM_2.5_, particulate matter measuring 2.5 μm or less.

^a^ Model 1 was adjusted for age, age squared, age cubed, sex, and race. Model 2 was adjusted for model 1 covariates and diabetes, chronic kidney disease, chronic obstructive pulmonary disease, heart failure, coronary artery disease, peripheral vascular disease, female sex, age squared, and heart failure as time-varying covariates. Model 3 was adjusted for model 2 covariates and census-level median household income, percentage with high school diploma, and percentage with some college education.

## Discussion

This cohort study of a large regional cohort of patients with AF found associations between air pollution exposure, measured by annual, residence-level PM_2.5_, and ischemic stroke. Individuals residing in residences with the highest quartile PM_2.5_ exposure had an approximately 20% greater risk of stroke compared with the lowest quartile. This association persisted after adjustment for demographic factors, comorbid conditions associated with stroke, and census-level socioeconomic factors of median income and educational attainment.

The study combined data from (1) residential estimates of PM_2.5_ using spatial saturation monitoring and LUR; (2) detailed EHR events from a large, regional health care system; and (3) census tract–level socioeconomic data to contribute to the substantive evidence of the public health toll of air pollution. Conducting the study in Allegheny County, Pennsylvania, was particularly important because of the region’s industrial history and rank as the seventh worst county nationally for annual PM_2.5_.^21,37^

The findings of the present study are consistent with studies^4,11^ that found that long-term exposures to air pollutants increase the risk of cardiovascular disease. These findings contribute new insights regarding longitudinal associations of residential estimates of PM_2.5_ and risk of stroke in individuals with AF. Prior literature^18,19,38,39,40,41^ on pollution and AF that examined short-term particulate matter exposure used central rather than residential-level pollutant monitoring, ascertained AF as a hospitalization event or by intracardiac device monitoring, or was limited by incomplete covariates. A meta-analysis^42^ of air pollution and AF identified significant heterogeneity (*I*^2^ = 0.65%) across studies. Another meta-analysis^20^ found that studies of PM_2.5_ and stroke have not focused specifically on participants with AF.

The inclusion of neighborhood-level social factors adds to the validity of the findings. Adjustment for neighborhood environment is crucial, given the documented contribution of socioeconomic position and social factors in cardiovascular health. In a large census data–based study, PM_2.5_ exposure was 1.5-fold higher in Black populations compared with White populations and 1.3-fold higher in those living below the poverty level vs above.^43^ Pollution exposure has also been associated with socioeconomic position as indicated by neighborhood racial and income distribution,^44,45^ and studies^46,47,48,49,50^ in community-based cohorts confirm that air pollution exacerbates racial disparities in health outcomes.

The dose-dependent result found in the present study suggests a biological mechanism underlying an association between progressively greater risk of ischemic stroke in individuals with AF. Furthermore, PM_2.5_ is associated with hypertension, diabetes, and heart failure^51,52^ and is proinflammatory and prothrombotic and increases stress hormone activation, all of which are related to AF and stroke.^53,54^ Elevated PM_2.5_ exposure and concomitant inflammation may contribute to thrombosis and precipitate cerebrovascular events. Additional pathways between particulate matter and thrombosis have been described.^4^ Another potential explanation of our findings is that the association between air pollution and ischemic stroke is independent of AF. That is, particulate matter exposure may be higher in individuals with elevated stroke risk that is a result of inequities such as limited access to preventive health services and treatment for stroke risk factors.

The association of PM_2.5_ with ischemic stroke found in the present study suggests that efforts to reduce pollution exposure may reduce the risk of stroke in high-risk populations with AF. Recognition of the adverse effects of air pollutants has already resulted in the Clean Air Act, legislation of air quality monitoring campaigns, regulatory enforcement to control emission sources, and air advisories.^37,55^ Government, professional society, and industry collaborations have developed initiatives to address pollution and reduce cardiovascular disease burden.^56^ This study provides additional evidence to support monitoring and advocacy for public health policy. Also, future studies to model the cost-effectiveness of PM_2.5_ reduction measures might aid in reducing stroke risk among individuals with AF.

### Strengths and Limitations

This study has strengths and limitations. One strength is that spatially refined estimates of long-term residence-based PM_2.5_ exposure were combined with extensive data on patient-level clinical risk factors and outcomes from EHR data. In addition, the fine-scale LUR surfaces of PM_2.5_ for Allegheny County allowed for more accurate exposure modeling than the traditionally used city- or countywide measures. These results may help in understanding the epidemiology of stroke in individuals with AF and add to the literature on air pollution exposure and ischemic stroke risk in AF.

This study has important limitations. First, there is potential for misclassification bias from several sources. Individuals may have had diagnoses outside the health care system or diagnoses may not have been captured by administrative coding. A second, fundamental limitation was the study’s inability to account for the duration of AF. The analysis was not designed to ascertain the date of incident AF; individuals may have been diagnosed before their entry into the EHR. However, although the study was not able to account for the duration of AF, consistent associations were observed between PM_2.5_ and ischemic stroke. Third, a mean annual residence-specific exposure to particulate matter was quantified using data from a monitoring campaign. The study was not able to account for individual-level exposures within the home or from vocational sources, travel, or change of residence; such assessments were beyond the scope of this analysis. Of note, the absence of tobacco exposure from the analysis is a potential limitation, given the strong association of tobacco with ischemic stroke. Accurate ascertainment of smoking status in the EHR is challenging^57^ and to be comprehensive requires measurement of tobacco strength, years of smoking, and secondhand smoking. Fourth, medical management to control AF or stroke risk factors, such as anticoagulation or management of diabetes or hypertension, was not captured. Fifth, residual confounding may contribute to the interpretation of the findings reported here. The models used in this study did not capture factors such as exercise, diet, primary prevention, and health care access.

## Conclusions

In this cohort study of a large regional health care system, consistent associations were observed between pollution exposure and ischemic stroke risk in individuals with AF. These results suggest a dose-dependent association between air pollution and stroke events and highlight the importance of air pollution to cardiovascular outcomes relevant to AF. Future research is needed to address the individual- and neighborhood-level factors that exacerbate the associations identified here. These results advance understanding of the costs of air pollution in terms of public health and strengthen the arguments for continued advocacy of efforts to curb pollution exposures.

## Notes

Supplement. **eTable 1.** *International Classification of Diseases Ninth Revision* (*ICD-9*) Code-Based Definitions **eTable 2.** Incidence of Ischemic Stroke per 1000 Person-Years by Residential Estimates of PM_2.5_ Quartile, Total Cohort and Stratified by Age (<75, ≥75 Years at Study Entry) and Sex **eFigure 1.** Flow Diagram Describing Participant Selection and Stepwise Exclusion From the Analysis **eFigure 2.** Ischemic Stroke Events Over Time by PM_2.5_ Quartile Stratified by Age <75 (A) and Age ≥75 Years (B) **eFigure 3.** Ischemic Stroke Events Over Time by PM_2.5_ Quartile Stratified by Sex
