# Supplementary material for: Association of Fine Particulate Matter and Risk of Stroke in Patients With Atrial Fibrillation
Source: JAMA Netw Open. 2020 Sep 15;3(9):e2011760. doi: 10.1001/jamanetworkopen.2020.11760 (PMC7492916; doi:10.1001/jamanetworkopen.2020.11760)
Supplement: Supplement. — eTable 1. International Classification of Diseases Ninth Revision (ICD-9) Code-Based Definitions eTable 2. Incidence of Ischemic Stroke per 1000 Person-Years by Residential Estimates of PM2.5 Quartile, Total Cohort and Stratified by Age (<75, ≥75 Years at Study Entry) and Sex eFigure 1. Flow Diagram Describing Participant Selection and Stepwise Exclusion From the Analysis eFigure 2. Ischemic Stroke Events Over Time by PM2.5 Quartile Stratified by Age <75 (A) and Age ≥75 Years (B) eFigure 3. Ischemic Stroke Events Over Time by PM2.5 Quartile Stratified by Sex [file jamanetwopen-e2011760-s001.pdf]

Supplementary Online Content

Rhinehart ZJ, Kinnee E, Essien UR, et al. Association of fine particulate matter and risk of stroke in patients with atrial fibrillation. *JAMA Netw Open*. 2020;3(9):e2011760.
doi:10.1001/jamanetworkopen.2020.11760

eTable 1. International Classification of Diseases Ninth Revision (ICD-9) Code-Based Definitions
eTable 2. Incidence of Ischemic Stroke per 1000 Person-years by Residential Estimates of $PM_{2.5}$ Quartile, Total Cohort and Stratified by Age (<75, ≥75 Years at Study Entry) and Sex
eFigure 1. Flow Diagram Describing Participant Selection and Stepwise Exclusion From the Analysis
eFigure 2. Ischemic Stroke Events Over Time by $PM_{2.5}$ Quartile Stratified by Age <75 (A) and Age ≥75 Years (B)
eFigure 3. Ischemic Stroke Events Over Time by $PM_{2.5}$ Quartile Stratified by Sex

This supplementary material has been provided by the authors to give readers additional information about their work.

© 2020 Rhinehart ZJ et al. *JAMA Network Open.*

**eTable 1.** International Classification of Diseases Ninth Revision (ICD-9) Code-Based Definitions	
**Medical Comorbidity**	**ICD-9 Codes**
Hemorrhagic stroke (hospitalization with any of the following as 1' diagnosis)	430, 431, 432, 432.1, 432.9
Ischemic or other stroke (hospitalization with any of the following as 1' diagnosis)	433.01, 433.11, 433.21, 433.31, 433.81, 433.91, 434.01, 434.11, 434.91, 436
TIA or amaurosis fugax (hospitalization with any of the following as 1' diagnosis)	435.0, 435.1, 435.2, 435.3, 435.8, 435.9, 362.34
Myocardial infarction hospitalization (hospitalization with any of the following as 1' diagnosis)	410.00, 410.01, 410.02, 410.10, 410.11, 410.12, 410.20, 410.21, 410.22, 410.30, 410.31, 410.32, 410.40, 410.41, 410.41, 410.42, 410.50, 410.51, 410.52, 410.60, 410.61, 410.62, 410.70, 410.71, 410.72, 410.80, 410.81, 410.82, 410.90, 410.91, 410.92
Heart failure hospitalization (hospitalization with any of the following as primary diagnosis)	402.01; 402.11; 402.91; 404.01; 404.03; 404.11; 404.13; 404.91; 404.93, 428, 428.0, 428.1, 428.2, 428.20, 428.21, 428.22, 428.23, 428.3, 428.30, 428.31, 428.32, 428.33, 428.4, 428.40, 428.41, 428.42, 428.43, 428.9
Hypertension	401, 401.0, 401.1, 401.9, 402, 402.0, 402.00, 402.01, 402.1, 402.10, 402.11, 402.9, 402.90, 402.91, 403, 403.0, 403.00, 403.01, 403.1, 403.10, 403.11, 403.9, 403.90, 403.91, 404, 404.0, 404.00, 404.01, 404.02, 404.03, 404.1, 404.10, 404.11, 404.12, 404.13, 404.9, 404.90, 404.91, 404.92, 404.93, 405, 405.0, 405.01, 405.09, 405.1, 405.11, 405.19, 405.9, 405.91, 405.99, 437.2
Diabetes	250, 250.0, 250.00, 250.01, 250.02, 250.03, 250.1, 250.10, 250.11, 250.12, 250.13, 250.2, 250.20, 250.21, 250.22, 250.23, 250.3, 250.30, 250.31, 250.32, 250.33, 250.4, 250.40, 250.41, 250.42, 250.43, 250.5, 250.50, 250.51, 250.52, 250.53, 250.6, 250.60, 250.61, 250.62, 250.63, 250.7, 250.70, 250.71, 250.72, 250.73, 250.8, 250.80, 250.81, 250.82, 250.83, 250.9, 250.90, 250.91, 250.92, 250.93
Heart failure	402.01, 402.11, 402.91, 404.01, 404.03, 404.13, 404.91, 404.93, 428, 428.0, 428.1, 428.2, 428.20, 428.21, 428.22, 428.23, 428.3, 428.30, 428.31, 428.32, 428.33, 428.4, 428.40, 428.41, 428.42, 428.43, 428.9
Coronary Artery Disease	414, 414.0, 414.00, 414.01, 414.02, 414.03, 414.04, 414.05, 414.06, 414.07414.2, 414.3, 414.4, 414.8, 414.9
Peripheral Vascular Disease	440.2, 440.20, 440.21, 440.22, 440.23, 440.24, 440.29, 440.4, 443.8, 443.81, 43.89, 443.9
History of hemorrhagic stroke (prior to date of AF ascertainment)	430, 431, 432, 432.1, 432.9
History of ischemic or other stroke (prior to date of AF ascertainment)	433.01, 433.11, 433.21, 433.31, 433.81, 433.91, 434.01, 434.11, 434.91, 436

© 2020 Rhinehart ZJ et al. *JAMA Network Open.*

History of TIA or amaurosis fugax (prior to date of AF ascertainment)	435.0, 435.1, 435.2, 435.3, 435.8, 435.9, 362.34
History of coronary artery disease or myocardial infarction	410.00, 410.01, 410.02, 410.10, 410.11, 410.12, 410.20, 410.21, 410.22, 410.30, 410.31, 410.32, 410.40, 410.41, 410.41, 410.42, 410.50, 410.51, 410.52, 410.60, 410.61, 410.62, 410.70, 410.71, 410.72, 410.80, 410.81, 410.82, 410.90, 410.91, 410.92, 412, 414.0, 414.2, 414.8, 414.9
History of heart failure	402.01; 402.11; 402.91; 404.01; 404.03; 404.11; 404.13; 404.91; 404.93, 428, 428.0, 428.1, 428.2, 428.20, 428.21, 428.22, 428.23, 428.3, 428.30, 428.31, 428.32, 428.33, 428.4, 428.40, 428.41, 428.42, 428.43, 428.9, 412, 414.00, 414.01, 414.02, 414.03, 414.04, 414.05, 414.06, 414.07, 414.2, 414.3,414.4, 414.8, 414.9

© 2020 Rhinehart ZJ et al. *JAMA Network Open.*

eTable 2. Incidence of Ischemic Stroke per 1000 Person-years by Residential Estimates of $PM_{2.5}$ Quartile, Total Cohort and Stratified by Age (<75, ≥75 Years at Study Entry) and Sex

	$PM_{2.5}$ Quartile			
Incidence*	10.30 (9.25-11.49)	12.16 (10.99-13.46)	12.51 (11.32-13.83)	15.64 (14.28-17.13)
Age				
<75	5.18 (4.22-6.37)	7.79 (6.52-9.30)	8.02 (6.77-9.50)	9.94 (8.51-11.62)
≥75	16.50 (14.53-18.74)	16.71 (14.77-18.92)	17.86 (15.78-20.22)	22.27 (19.91-22.92)
Sex				
Female	13.45 (11.65-15.52)	15.11 (13.28-17.19)	14.94 (13.14-16.99)	18.35 (16.36-20.60)
Male	7.88 (6.68-9.29)	9.22 (7.83-10.87)	10.00 (8.52-11.73)	12.54 (10.81-14.56)

*Adjusted for age and sex.

© 2020 Rhinehart ZJ et al. *JAMA Network Open.*

eFigure 1. Flow Diagram Describing Participant Selection and Stepwise Exclusion From the Analysis

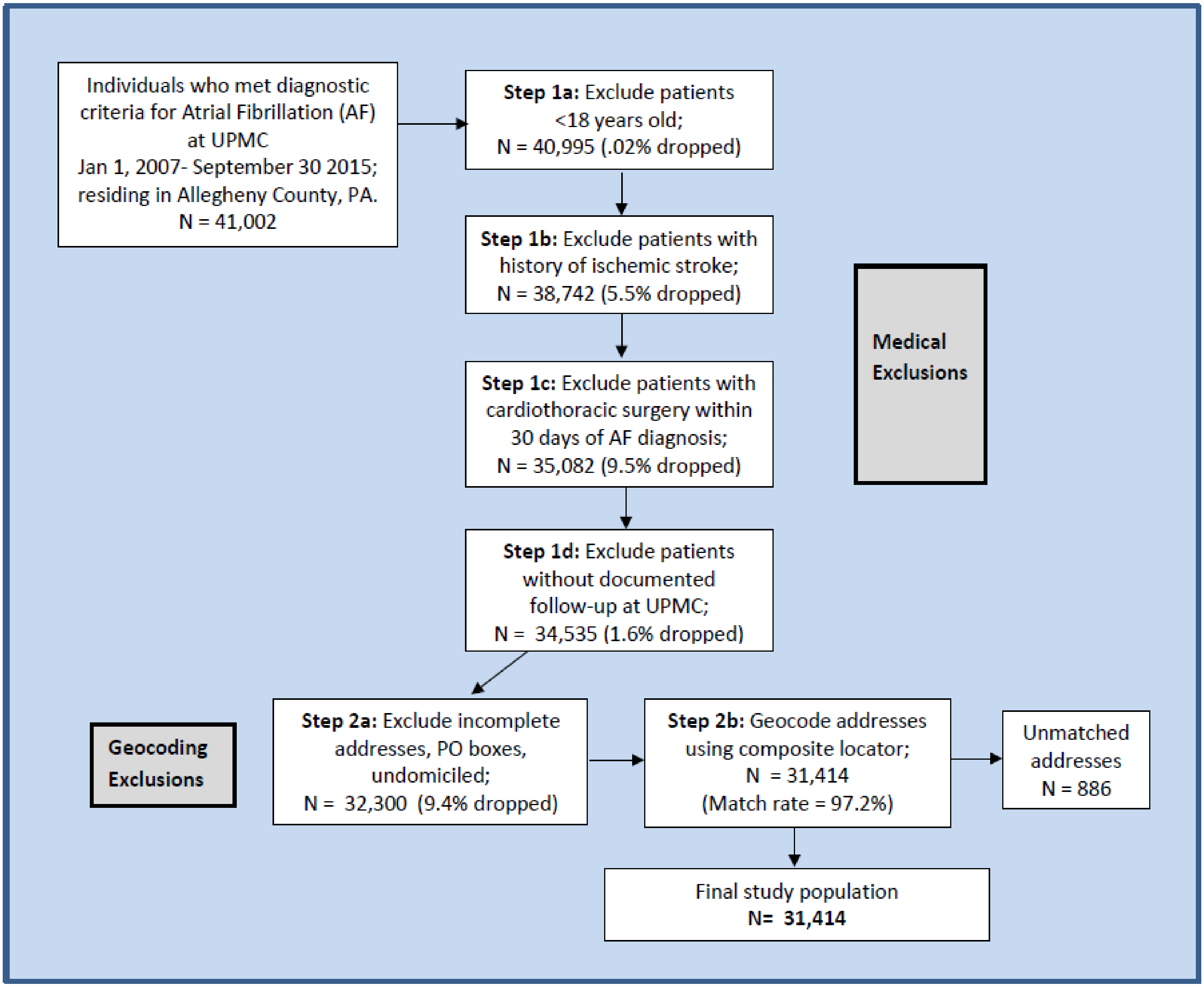

Following exclusions, there were 31,414 individuals included in the final analysis.

© 2020 Rhinehart ZJ et al. *JAMA Network Open.*

eFigure 2. Ischemic Stroke Events Over Time by $PM_{2.5}$ Quartile Stratified by Age <75 (A) and Age ≥75 Years (B)

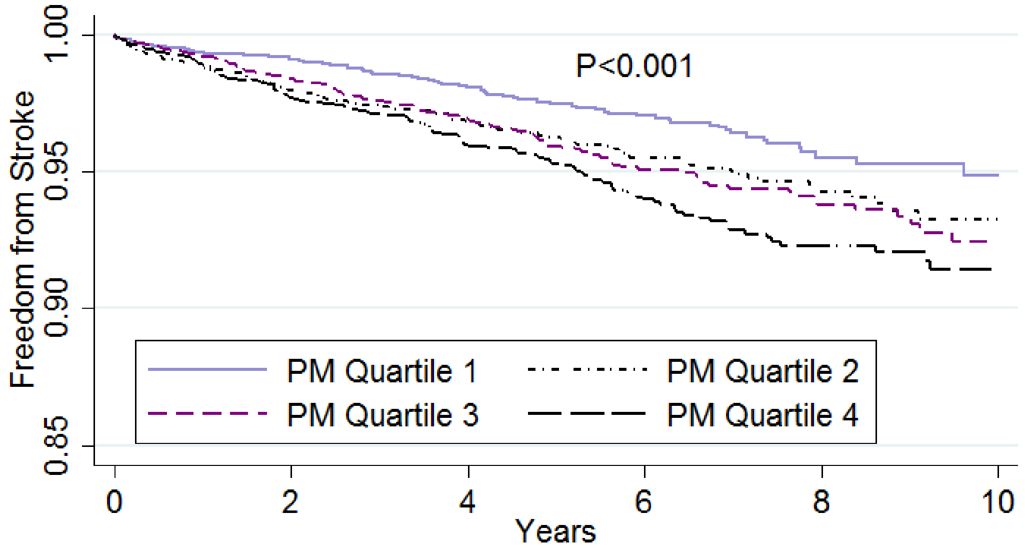

Number at risk	0	2	4	6	8	10
PM Quartile 1	3576	2942	2122	1286	524	143
PM Quartile 2	3330	2655	1830	1134	515	139
PM Quartile 3	3551	2855	1936	1177	576	170
PM Quartile 4	3572	2742	1806	1081	545	146

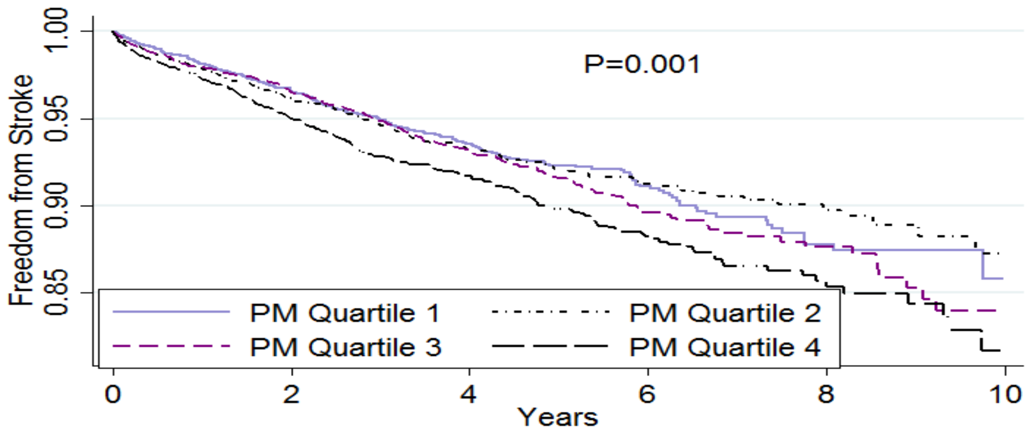

Number at risk	0	2	4	6	8	10
PM Quartile 1	4280	2762	1506	748	244	39
PM Quartile 2	4585	2916	1522	743	262	59
PM Quartile 3	4296	2741	1370	643	258	56
PM Quartile 4	4224	2724	1269	643	253	56

Both age-stratified analyses demonstrate the consistent association of the highest quartile of residential-level $PM_{2.5}$ with stroke events.

© 2020 Rhinehart ZJ et al. *JAMA Network Open.*

eFigure 3. Ischemic Stroke Events Over Time by $PM_{2.5}$ Quartile Stratified by Sex

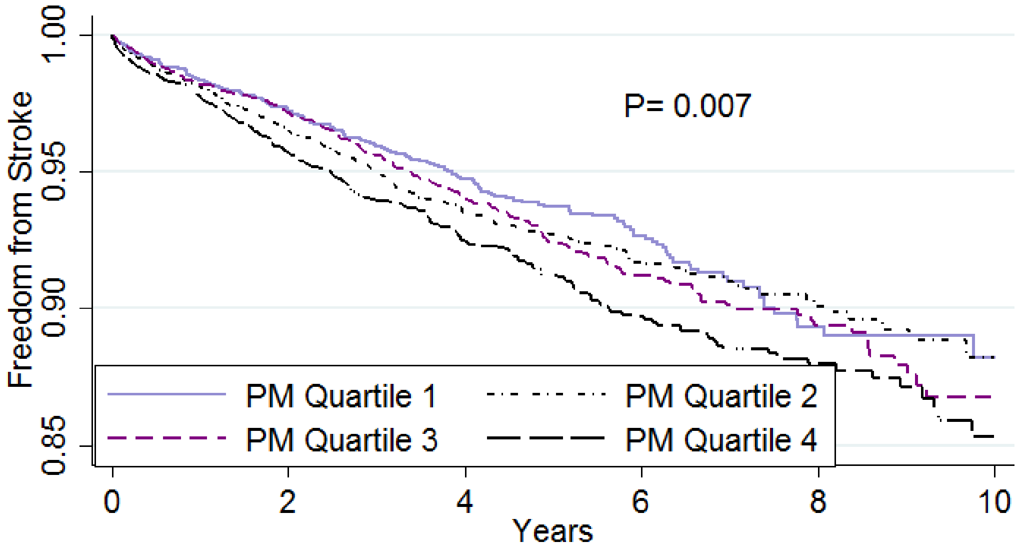

Number at risk

PM Quartile 1	3616	2531	1533	853	313	72
PM Quartile 2	3968	2770	1676	915	388	101
PM Quartile 3	4038	2869	1663	913	421	110
PM Quartile 4	4191	2937	1619	898	421	115

The survival curves by quartile of $PM_{2.5}$ in women (A) and men (B). While women in the cohort had greater incidence of strokes than men, the sex-stratified analyses demonstrate the consistent association of the highest quartile of residential-level $PM_{2.5}$ with stroke events in both men and women.

© 2020 Rhinehart ZJ et al. *JAMA Network Open.*

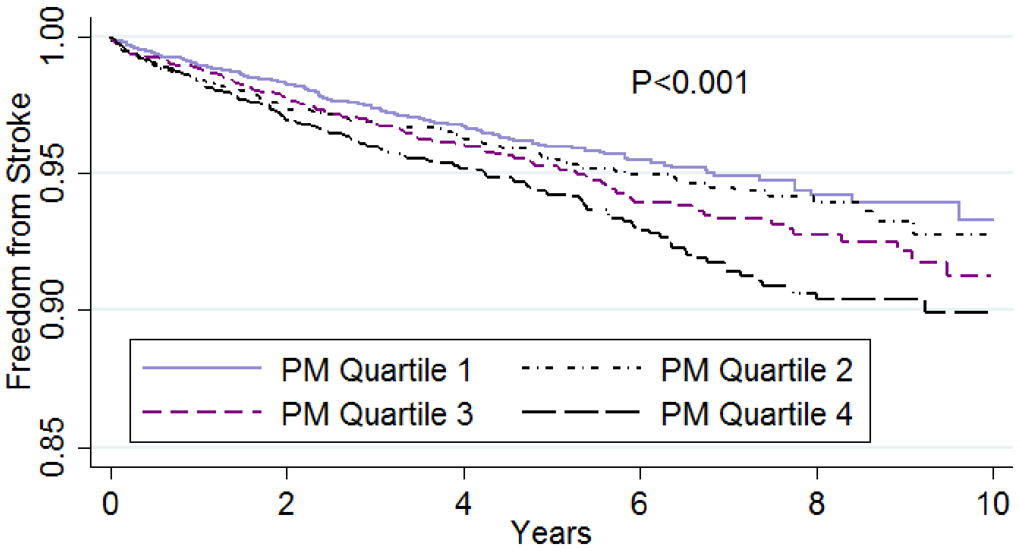

Number at risk

	0	2	4	6	8	10
PM Quartile 1	4240	3173	2095	1181	455	110
PM Quartile 2	3947	2801	1676	962	389	97
PM Quartile 3	3809	2727	1643	907	413	116
PM Quartile 4	3605	2529	1456	826	377	87

© 2020 Rhinehart ZJ et al. *JAMA Network Open.*
